# Impact of Lymph Node Ratio on Oncologic Outcomes in ypStage III Rectal Cancer Patients Treated with Neoadjuvant Chemoradiotherapy followed by Total Mesorectal Excision, and Postoperative Adjuvant Chemotherapy

**DOI:** 10.1371/journal.pone.0138728

**Published:** 2015-09-18

**Authors:** Taeryool Koo, Changhoon Song, Jae-Sung Kim, Kyubo Kim, Eui Kyu Chie, Sung-Bum Kang, Keun-Wook Lee, Jee Hyun Kim, Seung-Yong Jeong, Tae-You Kim

**Affiliations:** 1 Department of Radiation Oncology, Seoul National University College of Medicine, Seoul, Korea; 2 Department of Radiation Oncology, Seoul National University Bundang Hospital, Seongnam, Korea; 3 Department of Surgery, Seoul National University Bundang Hospital, Seongnam, Korea; 4 Department of Internal Medicine, Seoul National University Bundang Hospital, Seongnam, Korea; 5 Department of Surgery, Seoul National University College of Medicine, Seoul, Korea; 6 Department of Internal Medicine, Seoul National University College of Medicine, Seoul, Korea; University of Algarve, PORTUGAL

## Abstract

**Purpose:**

To evaluate the prognostic impact of the lymph node ratio (LNR) in ypStage III rectal cancer patients who were treated with neoadjuvant chemoradiotherapy (NCRT).

**Materials and Methods:**

We retrospectively reviewed the data of 638 consecutive patients who underwent NCRT followed by total mesorectal excision, and postoperative adjuvant chemotherapy for rectal cancer from 2004 to 2011. Of these, 125 patients were positive for lymph node (LN) metastasis and were analyzed in this study.

**Results:**

The median numbers of examined and metastatic LNs were 17 and 2, respectively, and the median LNR was 0.143 (range, 0.02–1). Median follow-up time was 55 months. In multivariate analyses, LNR was an independent prognostic factor for overall survival (OS) (hazard ratio [HR] 2.17, *p* = 0.041), disease-free survival (DFS) (HR 2.28, *p* = 0.005), and distant metastasis-free survival (DMFS) (HR 2.30, *p* = 0.010). When ypN1 patients were divided into low (low LNR ypN1 group) and high LNR (high LNR ypN1 group) according to a cut-off value of 0.152, the high LNR ypN1 group had poorer OS (*p* = 0.043) and DFS (*p* = 0.056) compared with the low LNR ypN1 group. And there were no differences between the high LNR ypN1 group and the ypN2 group in terms of the OS (*p* = 0.703) and DFS (*p* = 0.831).

**Conclusions:**

For ypN-positive rectal cancer patients, the LNR was a more effective prognostic marker than the ypN stage, circumferential resection margin, or tumor regression grade after NCRT, and could be used to discern the high-risk group among ypN1 patients.

## Introduction

The absolute number of metastatic lymph nodes (LNs) has been considered as an important prognostic factor for colorectal cancer [1–3]. In addition to the number of metastatic LNs, the number of examined LNs has been shown to be an independent prognostic factor for survival [4]. Meanwhile, neoadjuvant chemoradiotherapy (NCRT) followed by total mesorectal excision (TME) has become the treatment of choice for patients with LN-positive rectal cancer. This NCRT can result in a significant decrease in the number and size of examined LNs in the TME specimen [5]. Consequently, the number of examined LN is frequently below the recommended number of 12, regardless of the quality of TME and pathologic analysis. Hence, looking for a new method that can overcome the problem of overall LN retrieval was essential. The lymph node ratio (LNR), which is the ratio of metastatic to examined LNs, has recently been proposed as a prognostic factor in patients with stage III colorectal cancer [6, 7]. Recently, several studies have also evaluated the prognostic value of LNR in ypN-positive rectal cancer patients who were treated with NCRT [8–12]. However, all of these previous studies did not evaluate the impact of LNR with the consideration of tumor regression grade (TRG) or circumferential resection margin (CRM). As TRG [13, 14] and CRM [15, 16] are being regarded as important prognostic factors nowadays and the association of the LNR and the distant metastasis has not been fully evaluated in these studies, the contemporary prognostic impact of LNR among rectal cancer patients who underwent NCRT has yet to be proven.

For that reason, we assess the impact of the LNR with the consideration of TRG and CRM in predicting survival and recurrence in ypStage III rectal cancer patients after NCRT.

## Materials and Methods

### Patients

The institutional review boards (IRBs) of both Seoul National University Hospital (SNUH) and Seoul National University Bundang Hospital (SNUBH) approved this study. Because this study was carried out retrospectively, the IRBs waived the written informed consent from patients. And patient information was anonymized and de-identified prior to analysis. Between April 2004 and April 2011, 421 and 217 patients with rectal cancer received NCRT at the SNUH and the SNUBH, respectively. We selected patients who met the following inclusion criteria: pathologically confirmed primary rectal cancer, performance of TME, any ypT and ypN positivity, absence of distant metastasis at diagnosis, no history of other malignancies, and follow-up time of 12 months or more. A total of 125 patients remained and their medical records were retrospectively reviewed.

### Pre-treatment evaluation

At the initial staging work-up, digital rectal examination (DRE), colonoscopy, chest radiography, computed tomography (CT) of the abdomen and pelvis, and magnetic resonance imaging (MRI) of the pelvis were performed for all patients. If patients were suspected to have distant metastasis, MRI of the liver or positron emission tomography was carried out. Cancer stages were scored according to the American Joint Committee on Cancer (AJCC) Staging System, seventh edition. A tissue biopsy of the primary lesion was performed for pathologic confirmation. Complete blood counts (CBC) and liver function tests were included in the initial work-up. Carcinoembryonic antigen (CEA) levels were measured before NCRT and 4 weeks from the end of NCRT.

### Treatment

Preoperative radiotherapy consisted of whole pelvic irradiation and primary tumor boost. The whole pelvis was irradiated with a dose of 45 Gy at 1.8 Gy/fraction, and the primary tumor received boost radiotherapy with doses of 5.4 Gy at 1.8 Gy/fraction. All patients received preoperative radiotherapy in the prone position. The treatment method of radiotherapy has been described previously [17, 18]. During preoperative radiotherapy, all patients received chemotherapy concurrently. The chemotherapy regimens were 5-fluorouracil (5-FU; n = 67); 5-FU and leucovorin (FL) (n = 18); capecitabine (n = 33); capecitabine and oxaliplatin (n = 5); and cetuximab, irinotecan, and capecitabine (n = 2).

Curative resection with TME was generally performed 5 to 12 weeks (median: 7 weeks) after NCRT completion. Regarding surgery types, 116 patients (93%) underwent sphincter preserving surgery, and 9 patients (7%) underwent abdominoperineal resection. Adjuvant chemotherapy was performed for all patients. The regimens for adjuvant chemotherapy were FL (n = 79); capecitabine (n = 20); 5-FU, leucovorin, and oxaliplatin (FOLFOX) (n = 25); and capecitabine and oxaliplatin (n = 1).

### Pathologic examination

The entire TME specimens including mesorectal fat was serially sliced into 4-mm-thick sections and embedded in paraffin. TRG was assessed by Dworak system. The shortest distance from the outermost part of the tumor to the CRM was measured histologically. The CRM was considered positive, if tumor was located 1 mm or less from the surface of the specimen. All retrieved LNs were analyzed histologically. Metastatic LNs were defined as nodal tissue containing aggregates of tumor cells >0.2 mm in diameter.

### Follow-up

Patients were followed up every 3 months for the first 2 years, every 6 months for the next 3 years, and yearly thereafter. Follow-up evaluations included a clinical examination, DRE, CBC, liver function test, and assessment of CEA level at each visit. Chest x-ray and abdominal and pelvic CT scan were conducted every 6 months, and a colonoscopy was performed at 1, 3, and 5 years after surgery. Locoregional recurrence was defined as recurrence detected in the pelvis. Recurrence outside the pelvis was considered as distant metastasis.

### Statistical analysis

Overall survival (OS) was calculated as the time from the date of first treatment to the date of death. Disease-free survival (DFS) was defined as the time from the beginning of NCRT to the date of first disease recurrence, either locoregional failure or distant metastasis. Locoregional recurrence-free survival (LRRFS) and distant metastasis-free survival (DMFS) represented the interval from the first date of NCRT to the detection dates of locoregional recurrence and distant metastasis, respectively. Survival curves were generated using the Kaplan-Meier method. Log-rank tests were used to examine the univariate association between outcomes and the following clinicopathlogic factors: age, sex, type of surgery, pre-NCRT or post-NCRT CEA level, ypT stage, ypN stage, number of examined LNs, number of metastatic LNs, LNR, CRM, TRG, histologic grade, angiolymphatic invasion (ALI), venous invasion (VI), and perineural invasion (PNI). Multivariate Cox hazards analyses for OS, DFS, LRRFS, and DMFS were used to adjust comparisons for various factors including LNR, pN stage, CRM, and TRG.

LNR was defined as follows: the ratio of the number of metastatic LNs to the number of total examined LNs. The patients were categorized into two groups based on their LNR with a cut-off value of 0.152. This cut-off value was chosen with using Maxstat, the maximally selected rank method in R 2.13.0 (R Development Core Team, Vienna, Austria; http://www.R-project.org; [19]). All *p* values reported are two-sided, with *p* < 0.05 used to denote statistical significance.

## Results

### Characteristics of patients and tumors

The clinicopathologic features of the 125 patients are summarized in Table 1. The median age was 58 years (range, 33–83 years). There were 85 males and 40 females in this study. A sphincter preserving surgery was performed in 116 patients (93%). Median CEA levels were 3.0 ng/ml (range, 0.5–105.0 ng/ml) before NCRT, and 1.9 ng/ml (range, 0.5–27.7 ng/ml) after NCRT. Pathologic examination of the specimen led to the classification of 2 tumors as ypT0, 4 as ypT1, 17 as ypT2, 102 as ypT3, 97 as ypN1, and 28 as ypN2. The median number of examined LNs was 17 (range, 1–49) and median 2 LNs (range, 1–17) were pathologically involved. Median LNR value was 0.143 (range, 0.02–1). Involved CRM was observed in 22 patients (18%). Complete regression of primary tumor was observed only in 2 patients (2%).

**Table 1:** Patient and tumor characteristics (n = 125).

Characteristics		
Age (years)	58	(33–83)
Sex		
Male	85	(68)
Female	40	(32)
Type of surgery		
SPS	116	(93)
APR	9	(7)
Performance status		
0–1	123	(98)
2	2	(2)
Pre-NCRT CEA level (ng/mL)	3.0	(0.5–105.0)
≤5	84	(67)
>5	41	(33)
Post-NCRT CEA level (ng/mL)	1.9	(0.5–27.7)
≤5	114	(91)
>5	11	(9)	
cT stage			
2	9	(7)	
3	109	(87)	
4	7	(6)	
cN stage			
0	15	(12)	
1	78	(62)	
2	32	(26)	
ypT stage			
0	2	(2)	
1	4	(3)	
2	17	(14)	
3	102	(82)	
ypN stage			
1	97	(78)	
2	28	(22)	
No. of examined LNs	17	(1–49)	
No. of metastatic LNs	2	(1–17)	
1	56	(45)	
2–3	41	(33)	
≥4	28	(22)	
LNR	0.143	(0.02–1)	
CRM			
Negative	103	(82)	
Positive	22	(18)	
TRG			
0	5	(4)	
1	38	(30)	
2	61	(49)	
3	19	(15)	
4	2	(2)	
Histologic grade			
WD & MD	115	(92)	
PD & mucinous	10	(8)	
Angiolymphatic invasion			
Negative	94	(75)	
Positive	31	(25)	
Venous invasion			
Negative	108	(86)	
Positive	17	(14)	
Perineural invasion			
Negative	91	(73)	
Positive	34	(27)	

Values are presented as median (range) or number (%).

SPS, sphincter preserving surgery; APR, abdominoperineal resection; NCRT, neoadjuvant chemoradiotherapy; CEA, carcinoembryonic antigen; LN, lymph node; LNR, lymph node ratio; CRM, circumferential resection margin; TRG, tumor regression grade; WD, well differentiated; MD, moderately differentiated; PD, poorly differentiated.

### Survival outcomes and prognostic factor analysis

**Fig 1:**
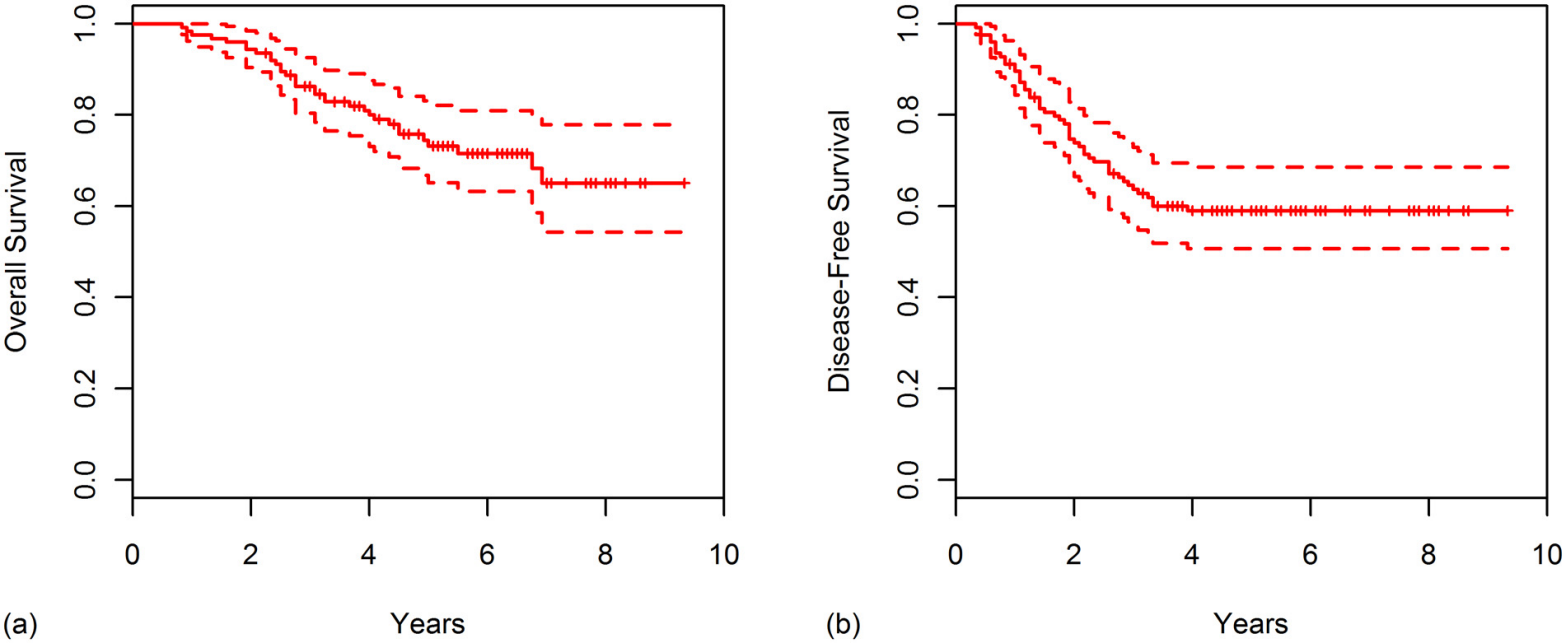
Kaplan-Meier curves for (A) overall survival and (B) disease-free survival for all patients.

The median follow-up time was 55 months (range, 8–112 months) for all patients, and 62 months (range, 27–112 months) for surviving patients. At 5 years, survival rates were as follows: OS (73.0%), DFS (58.9%), LRRFS (88.0%), and DMFS (65.0%). Survival curves for OS and DFS were plotted in Fig 1.

In univariate analysis (Table 2), the following factors were significantly associated with OS: type of surgery, ypT stage, LNR, TRG, ALI, VI, and PNI. Factors which were significantly associated with DFS were ypT stage, LNR, TRG, ALI, VI, and PNI. PNI was significantly associated with LRFFS. LNR, ALI, VI, and PNI were significantly associated with DMFS. The 5-year OS, DFS, LRRFS, and DMFS rate was 82.1%, 69.7%, 88.8%, and 74.2% for the patients with low LNR (LNR ≤0.152) and 56.6%, 40.2%, 82.3%, and 50.6% for the patients with high LNR (LNR >0.152), respectively. There were significant differences in OS (*p* = 0.006), DFS (*p* = 0.005), and DMFS (*p* = 0.005) rates between the patients with low LNR and high LNR.

**Table 2:** Univariate analysis according to clinicopathologic factors.

Variables		No.	5-yr OS (%)	*p*	5-yr DFS (%)	*p*	5-yr LRRFS (%)	*p*	5-yr DMFS (%)	*p*
Age (years)	≤60	72	78.1	0.193	59.0	0.929	88.3	0.662	66.0	0.926
	>60	53	66.1		58.5		86.7		62.7	
Sex	Male	85	73.2	0.591	60.4	0.692	89.0	0.564	64.1	0.901
	Female	40	72.9		56.1		86.0		66.5	
Type of surgery	SPS	116	75.3	0.001	60.4	0.095	88.3	0.618	65.9	0.180
	APR	9	44.4		40.0		83.3		53.3	
Pre-NCRT CEA level (ng/mL)	≤5	84	78.9	0.120	63.8	0.155	89.3	0.519	68.5	0.296
	>5	41	62.1		48.7		86.0		57.5	
Post-NCRT CEA level (ng/mL)	≤5	114	74.5	0.118	60.7	0.265	88.9	0.258	65.4	0.815
	>5	11	62.3		40.9		77.8		61.4	
ypT stage	ypT0–2	23	89.3	0.033	82.6	0.019	100	0.070	82.6	0.076
	ypT3	102	69.4		53.3		84.9		60.7	
ypN stage	ypN1	97	75.6	0.081	63.0	0.086	90.6	0.069	67.5	0.182
	ypN2	28	64.2		45.1		79.4		55.6	
No. of examined LNs	<12	37	78.0	0.504	55.0	0.504	90.4	0.754	56.7	0.277
	≥12	88	69.7		60.5		86.7		68.8	
No. of metastatic LNs	1	56	77.9	0.200	68.9	0.102	94.0	0.152	69.6	0.323
	2–3	41	73.4		55.1		86.8		64.5	
	≥4	28	64.2		45.1		79.4		55.6	
LNR	≤0.152	70	82.1	0.006	69.7	0.005	88.8	0.506	74.2	0.005
	>0.152	55	62.5		44.8		86.6		52.2	
CRM	Negative	103	74.5	0.234	61.3	0.174	88.8	0.373	65.8	0.536
	Positive	22	66.0		47.6		84.4		60.7	
TRG	0–2	104	68.6	0.017	54.2	0.040	86.2	0.314	61.8	0.122
	3–4	21	95.2		81.0		95.0		79.6	
Histologic grade	WD & MD	115	74.3	0.213	59.1	0.758	87.2	0.339	65.6	0.430
	PD & mucinous	10	58.3		56.3		100		56.3	
Angiolymphatic invasion	Negative	94	76.9	0.020	65.0	0.004	90.2	0.073	69.7	0.013
	Positive	31	61.5		40.3		80.4		50.1	
Venous invasion	Negative	108	78.1	<0.001	63.0	0.001	89.5	0.087	67.2	0.045
	Positive	17	42.5		31.9		76.6		49.9	
Perineural invasion	Negative	91	80.5	<0.001	68.6	<0.001	92.5	0.019	72.7	<0.001
	Positive	34	53.1		32.8		69.0		43.3	

OS, overall survival; DFS, disease-free survival; LRRFS, locoregional recurrence-free survival; DMFS, distant metastasis-free survival. Other abbreviations as in Table 1.

In multivariate analyses, LNR (hazard ratio [HR] 2.17, 95% confidence interval [CI] 1.03–4.57, *p* = 0.041), PNI (*p* = 0.002), and type of surgery (*p* = 0.004) were independent prognostic factors for OS (Table 3). Regarding DFS, LNR (HR 2.28, 95% CI 1.28–4.07, *p* = 0.005) and PNI (*p* < 0.001) were statistically significant. LNR (HR 2.30, 95% CI 1.23–4.32, *p* = 0.010) and PNI (*p* = 0.001) were also independent prognostic factors for DMFS.

**Table 3:** Multivariate analysis for evaluating prognostic factors influencing outcomes.

	OS		DFS		LRRFS		DMFS	
	*p*	HR (95% CI)	*p*	HR (95% CI)	*p*	HR (95% CI)	*p*	HR (95% CI)
LNR (>0.152)	0.041	2.17 (1.03–4.57)	0.005	2.28 (1.28–4.07)		–	0.010	2.30 (1.23–4.32)
Perineural invasion	0.002	2.96 (1.47–5.93)	<0.001	3.09 (1.75–5.46)	0.027	3.60 (1.15–11.24)	0.001	2.82 (1.51–5.25)
Type of surgery (APR)	0.004	3.91 (1.56–9.81)		–		–		–
TRG (3–4)	0.080	0.16 (0.02–1.24)		–		–		–
Pre-NCRT CEA level (>5 ng/mL)		–	0.073	1.70 (0.95–3.02)		–		–

HR, hazard ratio; CI, confidence interval. Other abbreviations as in Tables 1 and 2.

### Subgroup analysis of LNR according to the ypN stage

**Fig 2:**
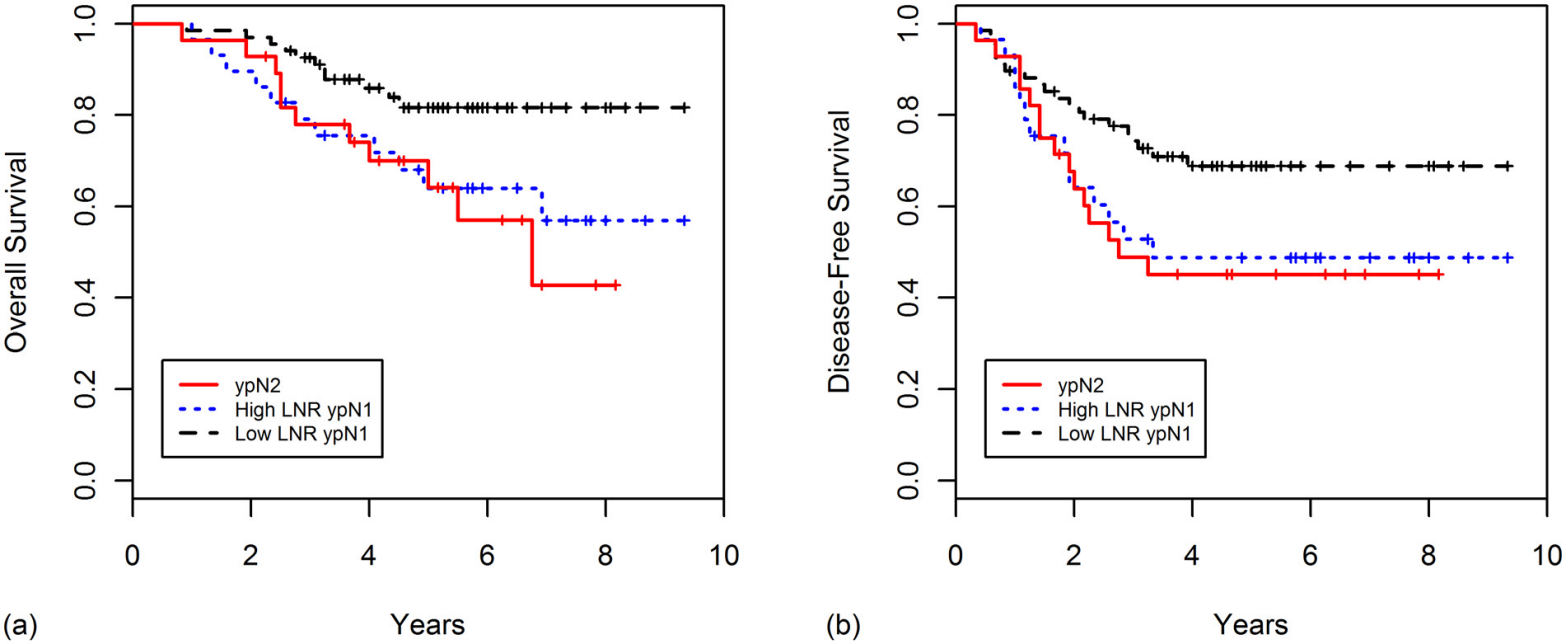
Kaplan-Meier curves for (A) overall survival and (B) disease-free survival according to ypN stage and lymph node ratio (low LNR ypN1, high LNR ypN1, and ypN2 group).

When ypN1 patients were divided into low (low LNR ypN1 group) and high LNR (high LNR ypN1 group) according to a cut-off value of 0.152, the high LNR ypN1 group had poorer OS (*p* = 0.043) and DFS (*p* = 0.056) compared with the low LNR ypN1 group. And there were no differences between the high LNR ypN1 group and the ypN2 group in terms of the OS (*p* = 0.703) and DFS (*p* = 0.831), indicating that the LNR has the superior stratification power over ypN stage. The survival curves for this classification are shown in Fig 2.

## Discussion

The main weakness of the number-based AJCC pN stage is that the prognostic accuracy can be profoundly influenced by the total number of LNs retrieved. The AJCC recommends a minimum of 12 LNs to ensure adequate LN retrieval and accurate staging. However, insufficient retrieval and examination of LNs are usual in clinical practice. In the circumstances of routine use of NCRT nowadays, patients often have < 12 LNs retrieved, despite the maintenance of all surgical standards [20]. This led to develop a new prognostic index, the LNR, that incorporates all the information about LNs in a single identifiable parameter. The LNR has been identified as a promising classification index in other malignancies including breast, pancreatic, and gastric cancer [21–23]. Furthermore, several studies have demonstrated that the LNR is superior to the pN stage in colorectal cancer [24–26]. In terms of rectal cancer, several studies have also analyzed the significance of LNR among patients who underwent adjuvant chemoradiotherapy [6, 7, 27] and NCRT [8–12]. Previous NCRT studies have shown that LNR is an independent prognostic factor for OS and DFS. Specifically, Kang *et al*. showed that 5-year OS rate was lower for patients with higher LNR (≤0.143, 57.1%; >0.143, 29.9%; *p* < 0.003) among 75 ypN-positive patients [12]. Lee *et al*. also demonstrated that 5-year OS rate was lower for patients with higher LNR (≤0.15, 90.3%; 0.16–0.3, 75.1%; >0.3, 45.1%; *p* < 0.003) among 154 ypN-positive patients [9]. The results of NCRT studies and the present study are summarized in Table 4.

**Table 4:** Previously reported lymph node ratio studies of rectal cancer patients who underwent preoperative chemoradiotherapy.

Author	Study years	No.	Proportion of preoperative CRT (%)	Proportion of adjuvant chemotherapy (%)	Median follow-up (months)	Median/mean examined LNs (range)	Median/mean positive LNs (range)	Cut-off value of LNR	Outcomes significantly associated with LNR
Kang *et al*.^12^	1990–2006	75	100	100	35	18 (5–80)	2 (1–79)	0.143	OS
Klos *et al*.^8^	1998–2008	281	100	67	42	12	NR	0.09 and 0.36	CSS
Lee *et al*.^9^	2001–2007	154	100	100	52	15 (3–46)	NR	0.15 and 0.3	OS and DFS
Nadoshan *et al*.^11^	1996–2007	128	100	49	39	10 (2–28)	6 (1–25)	0.2	OS, LRRFS, and DMFS
Madbouly *et al*.^10^	2006–2010	115	100	100	37	12 (5–25)	4 (1–19)	0.375	OS and DFS
Present study	2004–2011	125	100	100	55	17 (1–50)	2 (1–17)	0.152	OS, DFS, and DMFS

NR, not reported; CSS, cancer-specific survival. Other abbreviations as in Tables 1 and 2.

In the present study, the prognostic value of LNR was assessed in 125 ypN-positive rectal cancer patients treated with NCRT followed by total mesorectal excision and postoperative adjuvant chemotherapy. In a multivariate Cox model which also considered ypN stage, TRG, and CRM, the LNR was an independent prognostic factor and ypN stage was no longer significant. In addition, LNR has the potential to discriminate the high-risk group among patients with the same ypN stage. This finding is in line with that of Lee *et al*. [9] and Kang *et al* [12]. Adding the concept of LNR to the ypN stage will improve accuracy of predicting prognosis of rectal cancer. And the LNR can be used as a more useful indicator than the pN stage in terms of guiding the administration of intensified postoperative chemotherapy.

Intensified neoadjuvant chemotherapy including oxaliplatin or cetuximab, has failed to improve complete response or survival [28]. A recently reported prospective trial named ADORE compared the effect of postoperative adjuvant FOLFOX with adjuvant FL in rectal cancer patients treated with NCRT [29]. After 38 months of median follow-up time, the FOLFOX arm showed higher DFS than the FL arm (at 3 years, 71.6% vs. 62.9%; HR 0.657, 95% CI 0.434–0.994, *p* = 0.047). In particular, the benefits of FOLFOX were more significant for ypN1b patients (HR 0.356, 95% CI 0.132–0.960, *p* = 0.041) and ypN2 patients (HR 0.414, 95% CI 0.181–0.946, *p* = 0.037).

Meanwhile, the use of different cut-off values among reports is a limitation of LNR as a prognostic tool. Several methods have been used to determine cut-off values of LNR, including the receiver operating characteristic curve [10], mean or median value [11, 12], and atypical selections among several cut-off points [6, 8, 9, 27]. Our study used a maximal chi-square method in R software; this is meaningful in terms of minimizing subjectivity. However, our current study had several limitations, including its relatively small sample size and its retrospective design. A further large-scale prospective study is needed to determine the prognostic value of LNR and its optimal cut-off value in rectal cancer patients who underwent NCRT.

## Conclusion

For ypN-positive rectal cancer patients after NCRT followed by TME, LNR had more prognostic value for OS, DFS, and DMFS than the ypN stage, TRG or CRM. The LNR may be used to discern a high-risk group who might benefit from more intensive adjuvant chemotherapy.

## Supporting Information

S1 File Original clean data for analysis. (XLSX)
